# Serum uric acid and outcome in hospitalized elderly patients with chronic heart failure through the whole spectrum of ejection fraction phenotypes

**DOI:** 10.1186/s12872-023-03544-w

**Published:** 2023-11-30

**Authors:** Wei Yan, Hai-Ying Tang, Yong-Qiang Yang, Kun-Lun He

**Affiliations:** 1 Department of Geriatric Medicine, The First Affiliated Hospital of Soochow University, 188 Shizijie Road, Suzhou, 215006 Jiangsu China; 2 Department of Radiotherapy & Oncology, The Second Affiliated Hospital of Soochow University, San Xiang Road No. 1055, Suzhou, 215004 Jiangsu China; 3 Analysis Center of Big Data, Medical Innovation Research Division, Chinese PLA General Hospital, Beijing, China

**Keywords:** Chronic heart failure, Uric acid, Elderly patients

## Abstract

**Introduction:**

Elevated serum uric acid (SUA) levels have been associated with poor outcome in patients with heart failure (HF). Uric acid is associated with inflammation and microvascular dysfunction, which may differentially affect left ventricular ejection fraction (EF) phenotypes. We aimed to identify the role of SUA across EF phenotypes in hospitalized elderly patients with chronic HF.

**Methods:**

We analyzed 1355 elderly patients who were diagnosed with chronic HF. All patients had SUA levels measured within the first 24 h following admission. Patients with left ventricle EF were categorized as having HF with reduced EF (HFrEF, EF < 40%), HF with mid-range EF (HFmrEF, 40%≦LVEF ≦ 49%) or HF with preserved EF (HFpEF, LVEF ≥ 50%). Endpoints were cardiovascular death, HF rehospitalization, and their composite. The median follow-up period was 18 months.

**Results:**

Compared with the lowest SUA quartile, the highest SUA quartile was significantly associated with the endpoints (adjusted HR: 2.404, 95% CI: 1.178–4.906, P = 0.016; HR: 1.418, 95% CI: 1.021–1.971, P = 0.037; HR: 1.439, 95% CI: 1.049–1.972, P = 0.024, respectively). After model adjustment, a significant association of SUA with cardiovascular death and the composite endpoint persisted among HFrEF and HFmrEF patients in the highest SUA quartile (P < 0.05 for all).

**Conclusions:**

In hospitalized elderly patients with chronic HF, SUA is an independent predictor of adverse outcomes, which can be seen in HFrEF and HFmrEF patients.

## Introduction

Heart failure (HF), a clinical syndrome caused by structural or functional cardiac dysfunction, is highly prevalent in elderly people and is associated with high morbidity and mortality worldwide [1]. The prevalence of other diseases such as atrial fibrillation (AF) is also common in elderly patients with heart failure. According to left ventricular ejection fraction (EF), three different types of HF have been described: HF with reduced EF (HFrEF), HF with mid-range EF (HFmrEF) and HF with preserved EF (HFpEF) [2]. Serum uric acid (SUA), the end product of purine metabolism, has shown prognostic value in patients with various cardiovascular disorders [3, 4]. Exploring the prognostic value of SUA is of particular interest in the case of HF, as SUA levels are elevated in this condition [5]. Interestingly, high SUA levels in HF may reflect a specific metabolic change. The UA-forming enzyme xanthine-oxidase (XO) is overexpressed in HF patients, thus providing a mechanistic explanation for elevated SUA levels [6]. Indeed, high SUA concentrations are strongly associated with disease severity and mortality in HF [7, 8]. However, whether the association between SUA and prognosis holds true across HF elderly patients with different degrees of left ventricular function remains unknown. This study investigated whether the association between SUA and prognosis is altered when elderly patients are stratified according to LVEF phenotype.

## Materials and methods

### Patients

A total of 1355 outpatients (> 60 years old) admitted to the Department of Cardiology, Chinese PLA General Hospital (Beijing, China), with a clinical diagnosis of chronic HF between January 2011 and January 2014 were enrolled into the study. The diagnosis of chronic HF was based on standard guidelines such as the symptoms or signs, electrocardiograms, chest radiographs, and echocardiography [2]. Patients with left ventricle EF were categorized as having HFrEF (EF < 40%), HFmrEF (40%≦LVEF ≦ 49%) or HFpEF (LVEF ≥ 50%) [2]. Of the 1355 patients, 339 (25.0%), 375 (27.7%) and 641 (47.3%) patients were included in the HFrEF, HFmrEF and HFpEF groups, respectively. Venous blood samples were drawn in the morning after overnight fast. Serum D-dimer, natriuretic peptides, albumin, creatinine, sodium, cholesterol, hemoglobin, and SUA, were determined using standard laboratory procedures. Estimated glomerular filtration rate (eGFR) was calculated by the Modification of Diet in Renal Disease (MDRD) study Eq. [9]. Electronic medical records were used to obtain demographic variables, clinical data, laboratory values, echocardiographic parameters, and information on patients’ past medical history and medications. The study was approved by the Ethical Committee for Medical Research of Chinese PLA General Hospital and was conducted in accordance with the Helsinki Declaration. There were no specific exclusion criteria other than age > 60 years. After a median follow-up period of 18 months (interquartile range of 12 to 29 months), data on cardiovascular deaths and HF rehospitalization were obtained from the hospital medical records and telephone follow-up. Endpoints were cardiovascular death, HF rehospitalization and their composite.

### Statistical analysis

Data analysis was performed using the Statistical Package for the Social Sciences (SPSS 26.0). Continuous variables are described as the mean ± standard deviation. Categorical variables are expressed as frequencies and percentages. The continuous variables with normal and skewed distributions were compared using analysis of variance and Kruskal-Wallis tests, respectively. Categorical variables were compared using the chi-square test. A multivariate logistic regression model was used to assess the independent association with AF. The odds ratios (ORs) with 95% confidence intervals (CIs) were calculated. The Kaplan-Meier method was used to evaluate the cumulative survival rate for each outcome according to SUA quartiles. Differences between survival curves were analyzed using the log-rank test. Univariate and multivariate Cox regression models were used to analyze the association between the quartiles of SUA and outcomes. We recorded the multivariable hazard ratios (HRs) with 95% CIs. Univariate analysis for variables with a P value < 0.1 were considered for use in the multivariate model along with age and sex. The null hypothesis was rejects for values of p < 0.05.

## Results

### Patient demographics

A total of 1355 patients with baseline SUA concentrations available were included (Table 1). The patients were mostly male (60.2%), with a mean age of 72.55 ± 8.02 years. In the whole cohort, left ventricular EF averaged 48.05 ± 11.68%; specifically, there were 339 (25.0%) HFrEF, 375 (27.7%) HFmrEF, and 641 (47.3%) HFpEF patients. In the whole cohort, SUA levels averaged 372.09 ± 120.90 µmol/L. When analyzing SUA concentration relative to LVEF phenotypes, HFrEF patients had significantly higher values of SUA with a median SUA value of 412.96 ± 139.59µmol/L, whereas HFmrEF and HFpEF groups had similar values, 363.71 ± 117.55 and 355.16 ± 106.22µmol/L, respectively. The ranges of SUA for quartiles I-IV were ≤ 288.1µmol/L, 288.1–356.0µmol/L, 356.0-437.5µmol/L and > 437.5µmol/L, respectively. The highest SUA quartile was associated with a higher NYHA class, longer length of stay, impaired heart function, male sex, lower systolic blood pressure, lower sodium level, higher heart rate, higher D-dimer, higher N-terminal pro-brain natriuretic peptide level, higher rates of chronic kidney disease and more use of other diuretics, digoxin or spironolactone (Table 1). Most patients received therapy according to contemporary guidelines.

**Table 1:** Patients’ main characteristics

Variable	Whole cohort (N = 1355)	Q1 sUA ≤ 288.1 (N = 339)	Q2 sUA 288.1–356 (N = 339)	Q3 sUA 356-437.5 (N = 340)	Q4 sUA > 437.5 (N = 337)	P value
Age (years)	72.55 ± 8.02	72.60 ± 7.98	72.33 ± 7.73	72.69 ± 8.16	72.57 ± 8.22	0.946
Male (%)	60.2	48.1	55.2	68.2^ab^	69.4^ab^	< 0.001
Body mass index (kg/m^2^)	24.79 ± 3.83	24.30 ± 3.90	25.05 ± 3.73^a^	25.28 ± 3.76^a^	24.53 ± 3.88^c^	0.003
Systolic blood pressure (mmHg)	135.19 ± 20.44	137.55 ± 21.45	137.13 ± 20.16	135.74 ± 18.83	130.32 ± 20.51^abc^	< 0.001
Diastolic blood pressure (mmHg)	75.30 ± 12.32	75.74 ± 12.45	75.42 ± 12.33	75.54 ± 11.84	74.48 ± 12.68	0.560
Heart rate (bpm)	77.44 ± 15.63	77.86 ± 15.98	77.01 ± 14.91	75.43 ± 15.38^a^	79.47 ± 16.01^bc^	0.008
Hypertension (%)	74.6	72.0	74.9	73.8	77.7	0.374
Coronary artery disease (%)	77.7	78.5	79.4	77.6	75.4	0.636
Diabetes mellitus (%)	36.1	38.6	35.7	34.4	35.6	0.698
Atrial fibrillation (%)	51.1	50.1	52.8	49.1	52.5	0.724
Chronic kidney disease (%)	34.4	12.4	22.1^a^	37.1^ab^	66.2^abc^	< 0.001
D-dimer (ng/mL)	0.995 ± 1.679	0.935 ± 1.679	0.758 ± 1.171	0.797 ± 0.944	1.493 ± 2.430^abc^	< 0.001
NT-proBNP (pg/mL)	3199.13 ± 5299.74	2168.67 ± 3310.89	2189.35 ± 3007.43	2476.91 ± 4077.70	5980.14 ± 8117.76^abc^	< 0.001
Albumin (g/L)	39.21 ± 4.14	38.77 ± 4.45	39.83 ± 3.84^a^	39.63 ± 3.87^a^	38.61 ± 4.24^bc^	< 0.001
Creatinine (mg/dL)	97.22 ± 50.94	77.39 ± 38.28	86.44 ± 40.67^a^	96.20 ± 32.96^ab^	129.06 ± 68.48^abc^	< 0.001
Sodium (mmol/L)	140.55 ± 3.92	140.57 ± 4.54	140.74 ± 3.72	140.92 ± 3.29	139.96 ± 3.98^abc^	0.010
Cholesterol (mmol/L)	3.88 ± 0.98	3.83 ± 1.00	4.00 ± 0.96^a^	3.92 ± 0.98	3.79 ± 0.97^b^	0.021
Haemoglobin (g/L)	129.83 ± 19.81	127.59 ± 17.84	131.17 ± 17.73^a^	132.32 ± 19.56^a^	128.24 ± 23.31^c^	0.004
LVEF (%)	48.05 ± 11.68	50.09 ± 11.10	49.53 ± 11.13	48.14 ± 11.98^a^	44.44 ± 11.70^abc^	< 0.001
Left atrial diameter (mm)	41.67 ± 6.42	40.32 ± 6.46	41.29 ± 6.34^a^	41.73 ± 6.03^a^	43.35 ± 6.50^abc^	< 0.001
LVESD (mm)	37.98 ± 9.47	35.73 ± 8.00	37.14 ± 8.85^a^	38.24 ± 9.96^a^	40.83 ± 10.21^abc^	< 0.001
LVEDD (mm)	50.85 ± 8.82	48.73 ± 7.49	50.20 ± 8.17^a^	51.26 ± 9.14^a^	53.22 ± 9.73^abc^	< 0.001
ACEI or ARB (%)	60.3	58.4	59.6	62.6	60.5	0.713
Calcium-channel blockers (%)	44.5	44.5	45.1	44.1	44.2	0.993
Beta-blockers (%)	74.0	70.8	76.7	71.5	77.2	0.113
Other diuretics (%)	45.5	28.3	41.6^a^	41.2^a^	70.9^abc^	< 0.001
Digoxin (%)	28.7	19.2	25.4	27.9^a^	42.4^abc^	< 0.001
Nitrates (%)	63.8	64.9	63.1	63.5	63.5	0.966
Aspirin/clopidogrel (%)	77.3	76.7	79.4	82.1	70.9^bc^	0.004
Warfarin (%)	15.4	17.1	14.7	12.9	16.9	0.386
Statins (%)	78.4	82.3	84.1	79.1	68.0^abc^	< 0.001
Spironolactone (%)	45.9	31.9	41.3^a^	46.5^a^	64.1^abc^	< 0.001
Length of stay (days)	11.33 ± 8.56	11.15 ± 8.64	10.74 ± 7.93	10.51 ± 7.24	12.92 ± 10.04^abc^	0.001
NYHA class III/IV (%)	36.3	26.9	26.5	35.0^ab^	56.7^abc^	< 0.001

Data are expressed as mean ± SD or patient numbers (%). NT-proBNP, N-terminal pro-brain natriuretic peptide; LVEF, left ventricular ejection fraction; LVESD, left ventricular end systolic diameter; LVEDD, left ventricular end diastolic diameter; ACEI, angiotensin-converting enzyme inhibitor; ARB, angiotensin II receptor blocker; NYHA, New York Heart Association. Compared with Q1 group, ^a^P<0.05; Compared with Q2 group, ^b^P<0.05; Compared with Q3 group, ^c^P<0.05

### Factors associated to AF

Variables associated with AF were analyzed using multivariate logistic regression (Table 2). Multivariate regression analysis showed SUA (OR = 1.002, 95% CI 1.001 to 1.004, P = 0.004) was independently associated with AF in elderly individuals with CHF.

**Table 2:** Effects of Multiple Variables on atrial fibrillation in Multivariate Logistic Regression Analysis

Variable	Odds ratio	95% Confidence interval	P
Age (year)	1.056	1.033–1.079	< 0.001
Diastolic blood pressure (mmHg)	1.015	1.002–1.029	0.028
Heart rate (bpm)	1.016	1.005–1.028	0.005
Coronary artery disease (%)	0.475	0.300-0.751	0.001
Uric acid (µmol/L)	1.002	1.001–1.004	0.004
Creatinine (mg/dL)	0.996	0.993-1.000	0.043
Haemoglobin (g/L)	1.013	1.003–1.022	0.010
Left ventricular ejection fraction (%)	1.112	1.085–1.138	< 0.001
Left atrial diameter (mm)	1.136	1.101–1.172	< 0.001
Left ventricular end systolic diameter (mm)	1.015	1.002–1.029	0.028
Warfarin (%)	8.669	4.439–16.928	< 0.001

### SUA quartiles and outcome

**Fig. 1:**
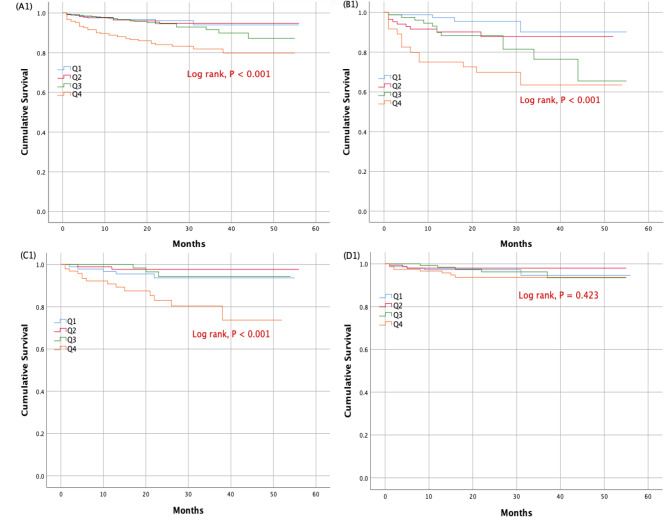
Kaplan-Meier survival curves for cardiovascular death according to uric acid quartiles in the whole population (**panel A1**), heart failure with reduced ejection fraction (**panel B1**), heart failure with mid-range ejection fraction (**panel C1**), and heart failure with preserved ejection fraction (**panel D1**)

**Fig. 2:**
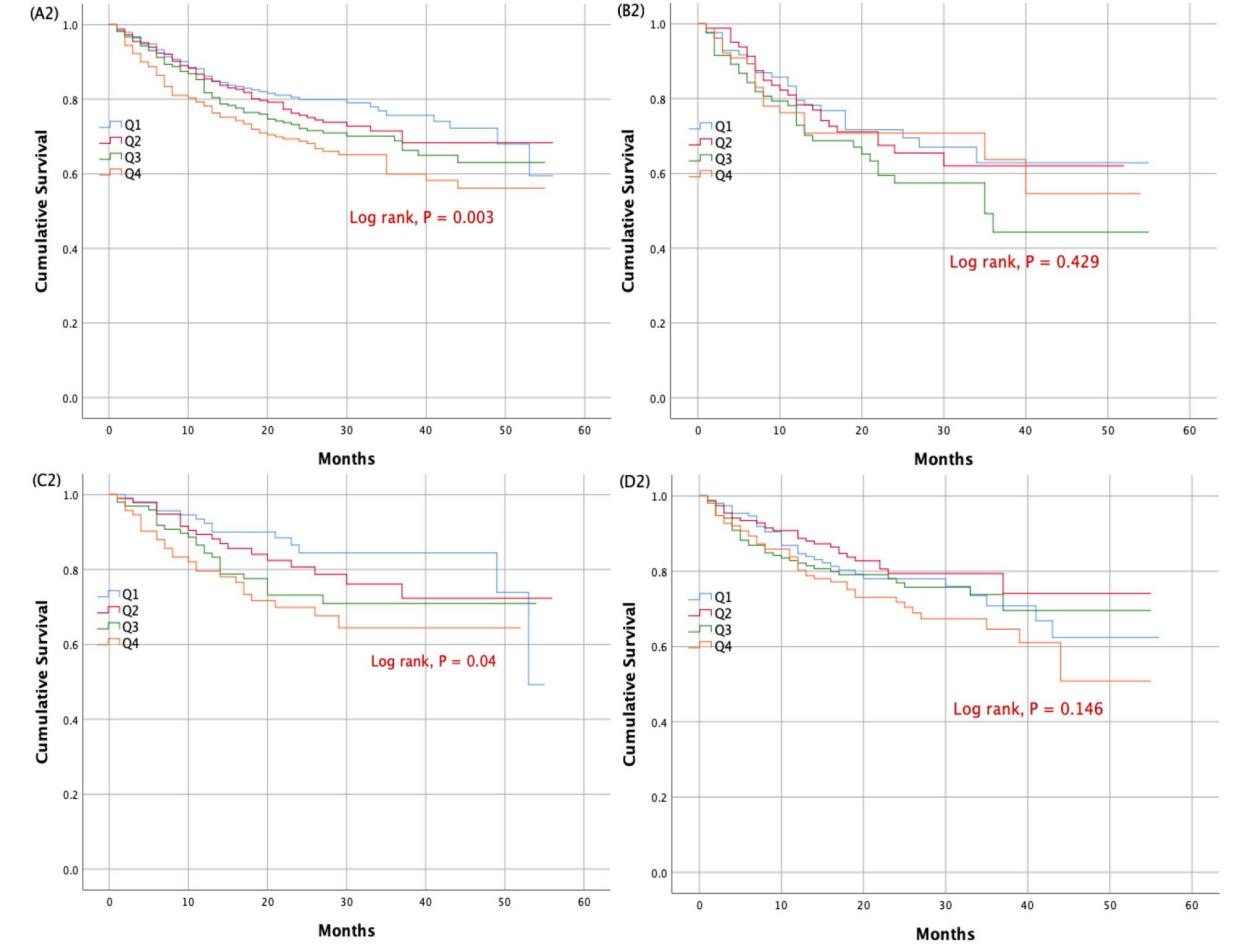
Kaplan-Meier survival curves for heart failure rehospitalization according to uric acid quartiles in the whole population (**panel A2**), heart failure with reduced ejection fraction (**panel B2**), heart failure with mid-range ejection fraction (**panel C2**), and heart failure with preserved ejection fraction (**panel D2**)

**Fig. 3:**
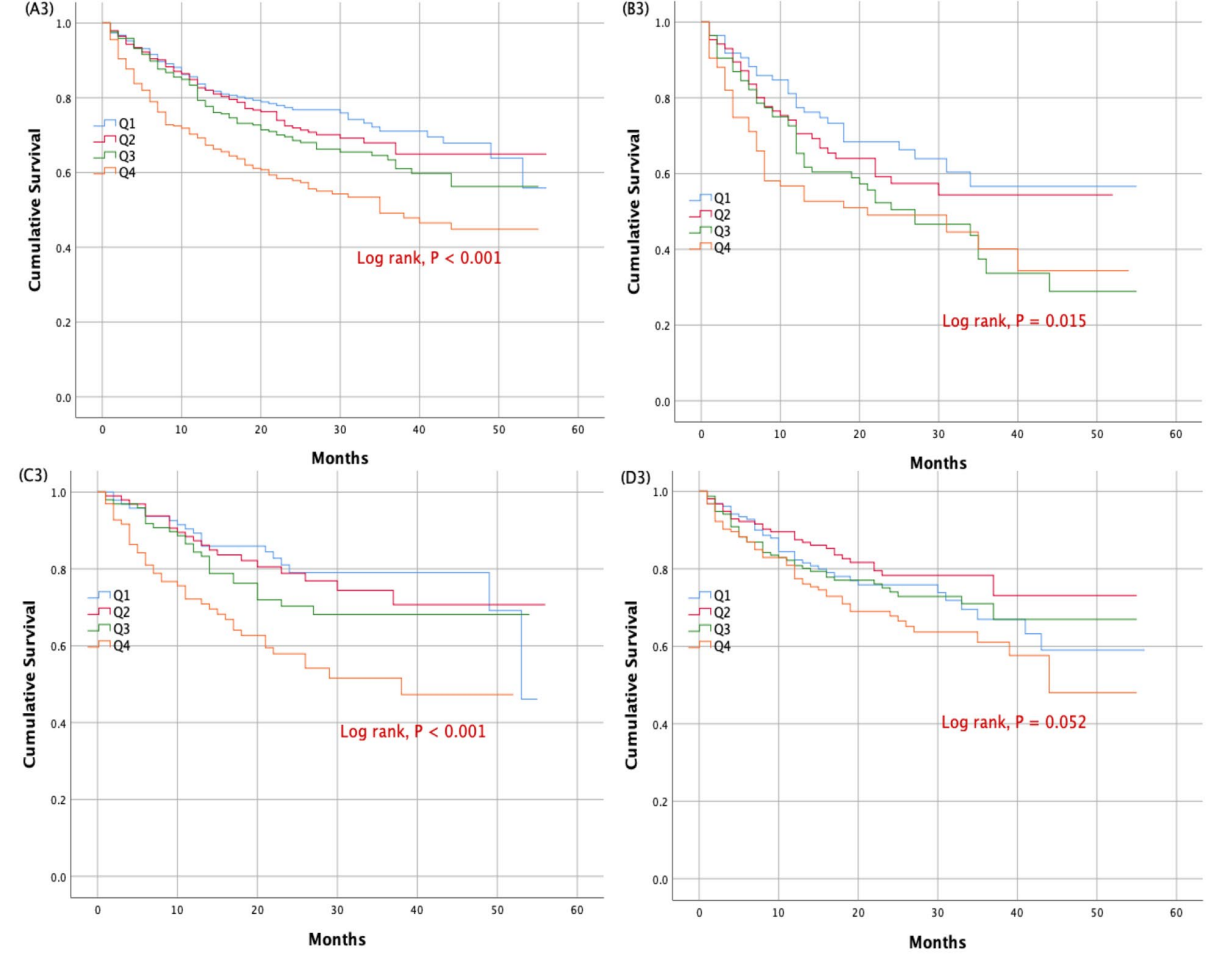
Kaplan-Meier survival curves for cardiovascular death or heart failure rehospitalization according to uric acid quartiles in the whole population (**panel A3**), heart failure with reduced ejection fraction (**panel B3**), heart failure with mid-range ejection fraction (**panel C3**), and heart failure with preserved ejection fraction (**panel D3**)

At 18 months, a total of 92 cardiovascular deaths (6.8%), 334 HF rehospitalization (24.6%), and 422 their composite (31.1%) occurred in the whole cohort. Subjects in the highest quartile displayed a significantly higher risk for cardiovascular deaths, HF rehospitalization and their composite than those in the lowest quartile (adjusted HR: 2.404, 95% CI: 1.178–4.906, P = 0.016; HR: 1.418, 95% CI: 1.021–1.971, P = 0.037; HR: 1.439, 95% CI: 1.049–1.972, P = 0.024, respectively) (Table 3). In the HFrEF cohort, at 18 months, there were 47 cardiovascular deaths (13.9%), 106 HF rehospitalization (31.3%) and 153 their composite (45.1%). In the HFrEF cohort, patients in the highest quartile showed a substantially increased risk for cardiovascular deaths and the composite prognosis compared to the lowest quartile (adjusted HR: 5.124, 95% CI: 1.457–18.012, P = 0.011; HR: 1.656, 95% CI: 1.017–2.697, P = 0.043, respectively) (Table 3). Among HFmrEF patients, at 18 months, there were 24 cardiovascular deaths (6.1%), 84 HF rehospitalization (21.4%) and 107 their composite (27.3%). In the HFmrEF cohort, an increase in the risk of cardiovascular deaths and the composite prognosis associated with the highest SUA quartile was also seen (adjusted HR: 3.923, 95% CI: 1.250–12.308, P = 0.019; HR: 2.504, 95% CI: 1.380–4.543, P = 0.003, respectively) (Table 3). The cumulative survival rate for each outcome due to heart failure as a function of SUA quartiles in the overall cohort and left ventricle EF subgroups of elderly patients admitted with chronic HF were determined using Kaplan-Meier curve analysis (Fig. 1, Figs. 2 and 3).

**Table 3:** Cardiovascular deaths, Heart failure rehospitalization and their composite by highest serum uric acid quartile in the whole population, and according to ejection fraction categories

Cardiovascular death	Serum Uric acid	N (%)	Multivariable HR	P value
Whole population	> 437.5	46/337 (13.6%)	2.404 (1.178–4.906)	0.016
HFrEF	> 492.0	22/84 (26.2%)	5.124 (1.457–18.012)	0.011
HFmrEF	> 431.4	14/98 (14.3%)	3.923 (1.250-12.308)	0.019
HFpEF	> 416.1	8/156 (5.1%)	0.902 (0.224–3.640)	0.885
**Heart failure rehospitalization**				
Whole population	> 437.5	99/337 (29.4%)	1.418 (1.021–1.971)	0.037
HFrEF	> 492.0	21/84 (25.0%)	0.887 (0.483–1.627)	0.698
HFmrEF	> 431.4	26/98 (26.5%)	1.801 (0.896–3.621)	0.099
HFpEF	> 416.1	45/156 (28.8%)	1.030 (0.622–1.705)	0.908
**Cardiovascular death or heart failure rehospitalization**				
Whole population	> 437.5	144/337 (42.7%)	1.439 (1.049–1.972)	0.024
HFrEF	> 492.0	43/84 (51.2%)	1.656 (1.017–2.697)	0.043
HFmrEF	> 431.4	40/98 (40.8%)	2.504 (1.380–4.543)	0.003
HFpEF	> 416.1	52/156 (33.3%)	0.913 (0.567–1.470)	0.709

## Discussion

In our study, elevated SUA concentrations indicated a substantially higher risk of future adverse outcomes in a large, contemporary cohort of elderly chronic HF inpatients, independent of other prognostic markers. Importantly, our data are the first to show that the adverse prognostic value of elevated SUA in elderly patients with chronic HF is not confined to HFrEF or HFpEF patients, as can be documented in other HF phenotypes as categorized based on left ventricle EF.

We discovered that SUA is an important factor associated to AF in elderly patients with chronic HF. Existing studies strongly suggest that hyperuricemia is independently associated with the increasing incidence of AF. Experimental and clinical data indicate that SUA is implicated in the pathophysiology of AF via activation of inflammation, oxidative stress, and fibrosis induced atrial remodeling. Briefly, atrial remodeling involves electrophysiological and structural abnormalities that promote the development of SUA induced AF. Also, SUA induced AF activates apoptosis and immune system [10]. The prevalence of AF is increasing as the population ages. HF and AF often coexist and each condition can promote the other, with an associated increase in overall morbidity and mortality [11]. Furthermore, physiological relations between AF and HF are multifactorial and causally intertwined [12]. So, a higher SUA was associated with an increased risk of AF which may make the prognosis of HF become worse in the elderly with chronic HF.

Our study found that SUA levels are associated with poor clinical outcomes in elderly patients with chronic HF, not only because of renal function or diuretic dose, but also because SUA itself has direct effects likely to be harmful in HF [13]. High SUA could also reflect increased xanthine oxidase activity, and this, in turn, might result in oxidative stress, which is thought to play a detrimental role in HF [14]. For example, SUA may have proinflammatory and proliferative actions and cause endothelial dysfunction [15]. Moreover, systemic inflammation and endothelial dysfunction possibly associated with SUA are also postulated to be consequences of HF [16]. Interestingly, recent findings indicate that elevated SUA is associated with higher cytokine levels and a greater inflammatory response, which suggests that inflammation may play an important role [17]. However, we did not measure cytokine concentrations in our study, and therefore, the precise role of elevated SUA in this context remains to be determined.

Our study found that in hospitalized elderly patients with chronic HF, SUA is an independent prognosticator of adverse outcomes, which can be seen in HFrEF and HFmrEF patients. SUA has been incorporated in HF risk scores [18], and the European HF guidelines report that hyperuricemia is common and is associated with worse prognosis in HFrEF patients [2]. HFmrEF is an intermediate clinical entity between HFrEF and HFpEF groups in some respects but is more like HFrEF in others, in particular with regard to the high prevalence of ischemic heart disease in these patients [19]. Elevated SUA is associated with poor outcomes in patients after MI complications due to reduced LV function, HF, or both. Xanthine oxidase is an enzyme that is crucially involved in myocardial degradation of ATP and purine and a source of reactive oxygen species. Previous work has shown that in HF, the xanthine oxidase inhibitor allopurinol reduces oxidative stress and ameliorates myocardial energy deficiency [20]. One may speculate that SUA is an indicator of the degree of myocardial energy status. Moreover, SUA has been identified as a DAMP (danger-associated molecular pattern molecule) that can trigger and maintain proinflammatory responses and may be related to cardiovascular morbidity [21]. Novel mechanistic insights into allopurinol have prompted clinical evaluation as a putative HF therapeutic.

### Study limitations

This study has several limitations. First, it was a single center and observational study with a limited number of patients. While follow-up lasted 18 months, it is possible that a longer follow-up would have allowed us to obtain further information so that the results should be considered to support the hypothesis and not to delineate cause and effect. Second, only baseline measurements of SUA levels were available, therefore, it was not possible to assess changes in SUA levels over time and to evaluate the implications of these changes on chronic HF outcomes. Third, unmeasured confounders may have not been considered in our analysis, and there was a difference in sample size among left ventricle EF strata, which could have affected risk estimate precision. Fourth, the proportion of more severe heart failure cases (NYHA heart failure class III/IV) was relatively low; thus, the results support the hypothesis, but future systematic studies are needed to confirm these findings.

## Conclusion

In a large, contemporary cohort of hospitalized elderly patients with chronic HF, high SUA levels were independently associated with worse outcome in cardiovascular death, HF rehospitalization and their composite over long-term follow-up. Our data also indicate for the first time that the adverse prognostic role of elevated SUA can be seen in elderly HFrEF and HFmrEF patients, which is associated with a high prevalence of ischemic heart disease in these patients. Considering that SUA is an easily available and inexpensive biomarker, it may represent a useful tool in the prognostic stratification of all patients with chronic HF.

## Data Availability

The datasets used and/or analyzed during the current study are available from the corresponding author on reasonable request.
